# Calcium-dependent protein kinases 2A involved in the growth of both asexual and sexual stages of *Cryptosporidium parvum*

**DOI:** 10.1371/journal.pntd.0013107

**Published:** 2025-05-28

**Authors:** Fanfan Shu, Yujin Huang, Fuxian Yang, Yaqiong Guo, Rui Xu, Lihua Xiao, Yaoyu Feng, Na Li

**Affiliations:** 1 State Key Laboratory for Animal Disease Control and Prevention, Center for Emerging and Zoonotic Diseases, South China Agricultural University, Guangzhou, Guangdong, China; 2 The Yunnan Key Laboratory of Veterinary Etiological Biology, College of Veterinary Medicine, Yunnan Agricultural University, Kunming, Yunnan, China; University of Ostrava: Ostravska univerzita, CZECHIA

## Abstract

**Background:**

*Cryptosporidium parvum* is a protozoan pathogen that causes moderate to severe diarrhea in both humans and animals. Calcium-dependent protein kinases (CDPKs) are attractive drug targets against cryptosporidiosis given their critical role in the life cycle of *Cryptosporidium* spp. and their absence in human and animal hosts.

**Methodology/principal findings:**

We used CRISPR-Cas9 technology to endogenously tag the *CpCDPK2A* gene in *C. parvum* IIdA20G1-HLJ strain with the hemagglutinin (HA) epitope and to delete the *CpCDPK2A* gene. An immunofluorescence assay was performed to localize the CpCDPK2A expression in the tagged strain and a luciferase assay was performed to compare growth rates of the tagged and deletion strains *in vitro*. Oocyst shedding, parasite load, villus length/crypt height ratio and survival of infected mice were used to evaluate the function of CpCDPK2A *in vivo*. The results revealed that CpCDPK2A was expressed in all the intracellular developmental stages, especially in the motile stages of sporozoites and merozoites. While CpCDPK2A is dispensable, deletion of the gene significantly reduced the growth of late asexual and sexual stages *in vitro*. In an interferon-γ knockout mouse model, gene deletion of *CpCDPK2A* reduced oocyst shedding by 25-fold and increased survival of infected mice.

**Conclusions/significance:**

These observations suggest that CpCDPK2A may contribute to both asexual and sexual replication of *C. parvum* and may be a potential target to block the transmission of this important zoonotic pathogen.

## Author summary

Cryptosporidiosis is a major cause of diarrhea in children and young animals, particularly in low- and middle-income countries. Despite its impact, effective drugs or vaccines are lacking. Calcium-dependent protein kinases (CDPKs) in *Cryptosporidium parvum* are promising therapeutic targets due to their unique structures. Whole genome sequencing and comparative genomic analyses have identified at least eight CpCDPKs in *C. parvum*. Our study focuses on CpCDPK2A, which is expressed in all intracellular stages and plays a crucial role in both asexual and sexual replication. Deletion of CpCDPK2A significantly reduces infection intensity and pathogenicity without affecting host cell invasion. These findings suggest that CpCDPK2A may be a viable target for the development of prevention strategies against cryptosporidiosis. Further research is needed to explore its vaccine potential and action mechanism.

## Introduction

Cryptosporidiosis is a leading cause of diarrhea in humans and various animals [1]. It causes moderate-to-severe diarrhea in children under two years of age in low and middle-income countries, and foodborne, waterborne and animal-contact associated outbreaks of cryptosporidiosis in high-income countries [2,3]. Despite its clinical and public health importance, there are no effective vaccines against cryptosporidiosis. The only approved drug for the treatment of cryptosporidiosis by the U.S. Food and Drug Administration, nitazoxanide, is ineffective in malnourished children and immunocompromised patients [4]. This has been attributed in part to the poor understanding of mechanisms of *Cryptosporidium* development [5].

Calcium is an important second messenger involved in numerous signaling cascades in the asexual and sexual development of apicomplexan parasites [6]. Among various calcium receptors, calcium-dependent protein kinases (CDPKs) are involved in many physiological processes [7]. Because CDPKs have unique 3D structures and are absent in mammals, they are considered to be ideal targets against cryptosporidiosis [9]. To date, whole genome sequencing and RNA-seq analysis have identified at least 11 CDPKs in *C. parvum* (CDPKs) [8]. These CpCDPKs have different gene expression patterns in various development stages of *C. parvum* [8]. The *CpCDPK1* gene is expressed at all life stages and has been identified as a valid drug target [8–9]. Several inhibitors of CpCDPK1 have shown promise in the treatment of cryptosporidiosis in experimental animals [9].

Recently, the functions of a few CpCDPKs have been characterized using genetic manipulation tools. Gene-editing studies using CRISPR-Cas9 have shown that CpCDPK1 is essential for the survival of *C. parvum*, with high expression in mature meronts [10]. Inhibition of CpCDPK1 expression significantly reduces parasite load *in vitro*, highlighting its critical role in early asexual replication [11]. In contrast, CpCDPK5 was found to be specifically involved in the sexual development, where deletion of CpCDPK5 impairs male gamete egress and reduces parasite virulence, disease severity and oocyst shedding of *C. parvum* [12]. Some other CpCDPKs, such as CpCDPK2A, CpCDPK3, CpCDPK4, CpCDPK6, and CpCDPK9, have been tentatively implicated in host cell invasion and subsequent intracellular growth, which need further verification using genetic manipulation tools [13–16].

In this study, we further characterized CpCDPK2A using CRISPR-Cas9 technology. Results of the study indicate that CpCDPK2A is expressed in all intracellular stages and plays an important role in both asexual and sexual replication of *C. parvum*, and deletion of the *CpCDPK2A* gene leads to significant reduction in infection intensity and pathogenicity of the parasite. These findings suggest that CpCDPK2A may act as a multi-stage regulator of parasite development and is a viable target for the development of prevention strategies against cryptosporidiosis.

## Results

### Epitope tagging of the *CpCDPK2A* gene

**Fig 1:**
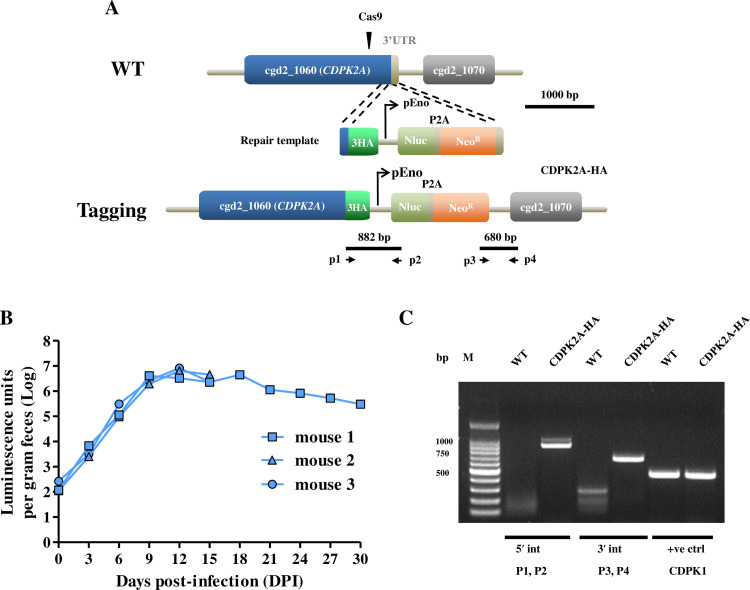
Endogenous tagging of the *CpCDPK2A* gene in *Cryptosporidium parvum.* (A) Diagram illustrating the strategy for adding the triple hemagglutinin epitope tag (3HA) and Nluc-P2A-Neo^R^ cassette to the C-terminus of *CpCDPK2A*. The single Cas9 guide sequence targets the kinase domain of *CpCDPK2A*. P2A is the split peptide, while P1-P4 represent primers used to verify the correct insertion events. (B) Fecal luminescence (log_10_) in GKO mice infected with the transgenic CDPK2A-HA strain. Each blue stain represents data from a single mouse. (C) Confirmation of the correct integration events at the 5′ (P1 and P2) and 3′ (P3 and P4) ends of the insertion using PCR analysis of genomic DNA extracted from wild-type (WT) and transgenic CDPK2A-HA parasites.

To understand the subcellular localization of CpCDPK2A expression, a 3 × HA epitope tag was introduced into the C-terminus of the *CpCDPK2A* gene by CRISPR-Cas9 (Fig 1A). Two mice infected with the CDPK2A-HA strain died at 15 DPI in agreement with the high pathogenicity of the wild-type strain (Fig 1B). Diagnostic PCR analysis of fecal DNA showed the correct insertion of the 3HA-Nluc-Neo^r^ cassette into the *CpCDPK2A* locus (Fig 1C). Thus, endogenous tagging was successful and did not attenuate the virulence of the wild-type *C. parvum* strain.

### Expression of CpCDPK2A in developmental stages of *C. parvum*

**Fig 2:**
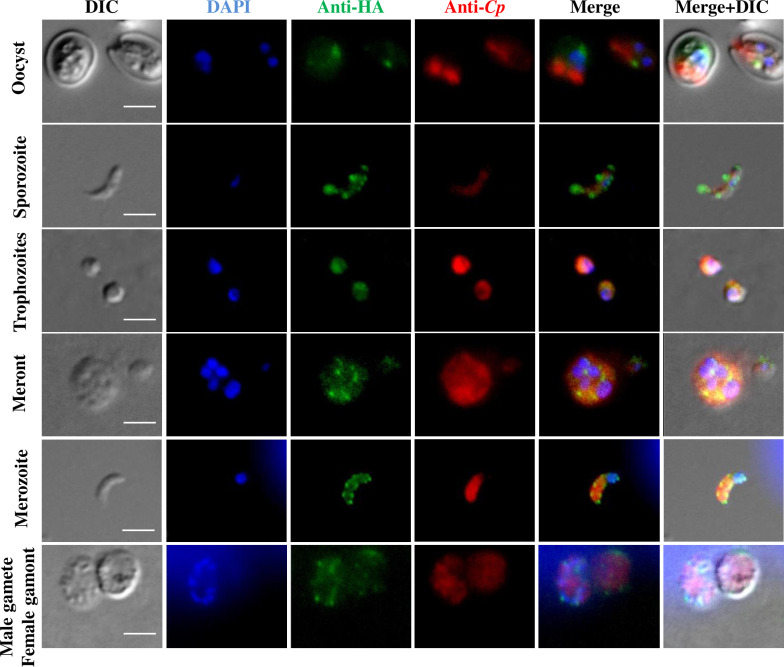
Expression of CpCDPK2A in oocysts, sporozoites, trophozoites, meronts, merozoites, and male gametes and female gamonts as indicated by immunofluorescence examination of the transgenic CDPK2A-HA strain of *Cryptosporidium parvum.*

To localize the CpCDPK2A expression, *in vitro* stages of the transfected strains were examined by IFA using antibodies against the HA epitope. CpCDPK2A was expressed in oocysts, developing trophozoites, mature meronts, and both male gametes and female gamonts (Fig 2). The expression of the protein in trophozoites and female gamonts was localized in the cytoplasm of *C. parvum*, while its expression in oocysts, meronts, and male gametes appeared to be confined to surface of sporozoites, merozoites, and male gametes within these stages, respectively. In most stages, CpCDPK2A accumulated unevenly on the surface. These results suggest that CpCDPK2A might have multiple functions in various developmental stages of *C. parvum*.

The transgenic parasites were stained with anti-HA antibodies (in green) and anti-*Cryptosporidium* antibodies (in red), with the nuclei being counter-stained with DAPI (in blue). Scale bars = 2 μM.

### Dispensable nature of CpCDPK2A for *C. parvum* growth

**Fig 3:**
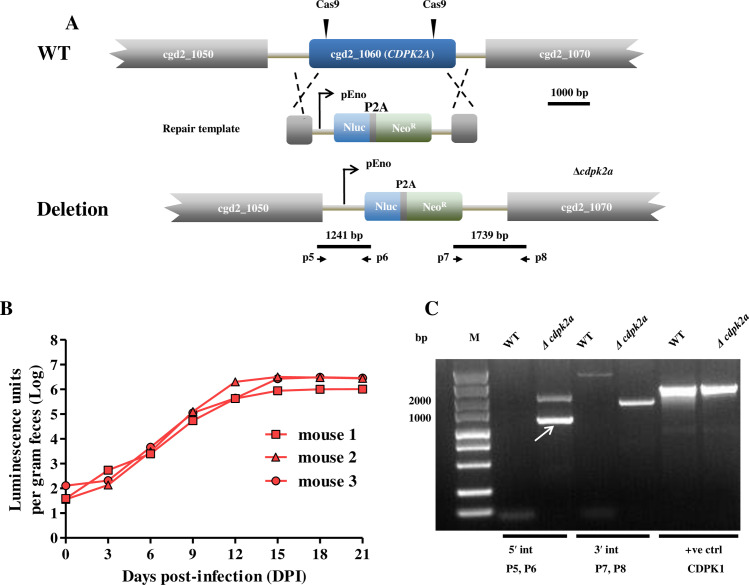
Construction of the ***Δcdpk2a* strain of *Cryptosporidium parvum*.** (A) Strategy for the replacement of the *CpCDPK2A* gene by a Nluc-P2A-Neo^R^ cassette using CRISPR-Cas9-mediated homologous recombination. The double Cas9 guide sequences targeted the kinase domain of *CpCDPK2A*. P5-P8 are primers used to verify the correct insertion events. (B) Fecal luminescence (log_10_) in mice infected with the *Δcdpk2a* strain. Each redline represents data from a single mouse. (C) Confirmation of the correct integration events at the 5′ (P5 and P6) and 3′ (P7 and P8) ends of the insertion by PCR analysis of genomic DNA extracted from wild-type (WT) and transgenic *Δcdpk2a* parasites. The control product is amplified form the *CpCDPK1* locus.

**Fig 4:**
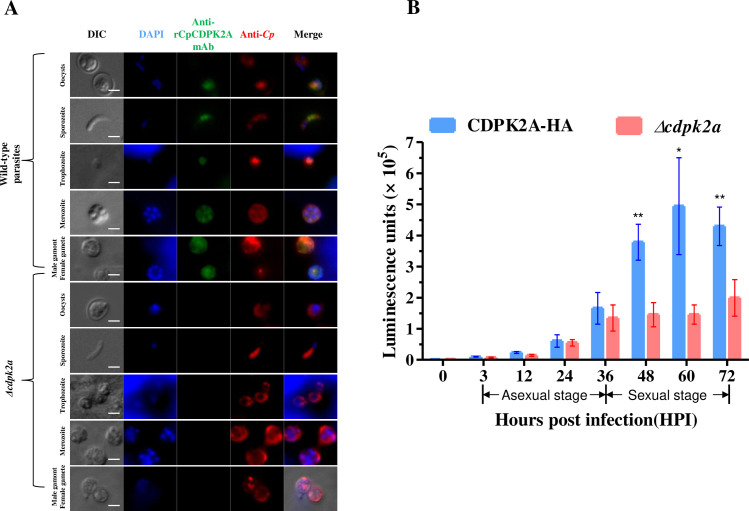
Verification of the *CpCDPK2A* gene disruption using immunofluorescence and effect of the gene deletion on the growth of *Cryptosporidium parvum* in HCT-8 cells. (A) Verification of *CpCDPK2A* disruption using a monoclonal antibody (mAb) against recombinant CpCDPK2A (rCpCDPK2A). Wild type (WT) and *Δcdpk2a* parasites were stained with anti-rCpCDPK2A antibodies (in green) and anti-*Cryptosporidium* antibodies (in red), while the nuclei were counter-stained with DAPI (in blue). Scale bars = 2 μM. (B) Luminescence of HCT-8 cell cultures infected with the CDPK2A-HA (in blue) and *Δcdpk2a* (in red) strains at different time (n = 4). * *p* = 0.0201 at 60 HPI, ** *p* = 0.0012 at 48 HPI, *p* = 0.0089 at 72 HPI. Statistical significance was assessed with an unpaired *t* test.

CRISPR-Cas9 technique was used to delete the *CpCDPK2A* gene in *C. parvum* using double guide sequences recognizing the N- and C-termini of the gene, with the gene completely replaced by a Nluc-Neo^r^ cassette containing homologous arms (Fig 3A). In GKO mice (*n* = 3) inoculated with the transfected sporozoites, fecal luminescence signal increased from 6 to 21 DPI (Fig 3B). PCR analysis of fecal materials confirmed that the native *CpCDPK2A* gene was replaced by the Nluc-Neo^r^ cassette (Fig 3C). In addition, both polyclonal and monoclonal antibodies against rCpCDPK2A failed in recognizing the *Δcdpk2a* parasite while they easily reacted with wild-type parasites (Figs 4A and S1). Therefore, the *CpCDPK2A* gene was successfully deleted in the *C. parvum* genome without obvious lethal effect on the pathogen.

### Involvement of CpCDPK2A in asexual and sexual development of *C. parvum*

A luciferase assay was used to compare the growth rates of *Δcdpk2a* and CDPK2A-HA strains *in vitro* (Fig 4B). The luminescence activities of *Δcdpk2a* and CDPK2A-HA parasites were similar prior to 24 HPI of the HCT-8 cell culture. At 36 HPI, the value in cultures infected with *Δcdpk2a* was lower than those infected with CDPK2A-HA, although the difference was not significant (*p* = 0.5171). Afterwards, luciferase activities in the *Δcdpk2a* strain remained constant until the termination of the culture at 72 HPI, and were significantly lower than those in the CDPK2A-HA strain (*p* = 0.0012, 0.0201, and 0.0089 at 48, 60, and 72 HPI, respectively). These results indicate that CpCDPK2A is involved in both asexual and sexual development of the parasite (Fig 4B).

### Reduced pathogenicity of *Δcdpk2a* strain in GKO mice

**Fig 5:**
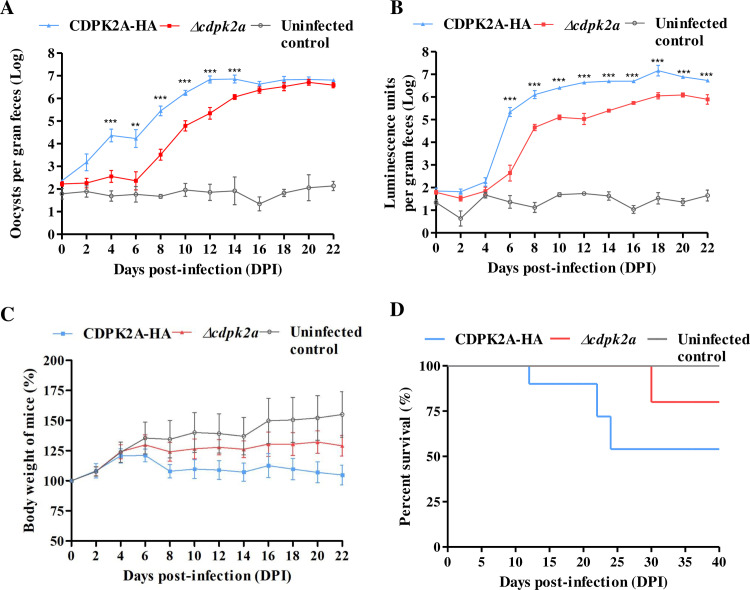
Effects of the *CpCDPK2A* gene deletion on oocyst shedding and pathogenicity of *Cryptosporidium parvum* in GKO mice. (A-B) Differences in parasite load in feces of GKO mice infected with the CDPK2A-HA and *Δcdpk2a* strains (1,000 oocysts/mouse) as indicated by oocysts per gram of feces (A) and fecal luciferase activity (B). Mice demonstrated ~25-fold reduction in infection intensity after the deletion of *CpCDPK2A* (*n* = 6 mice per group; ** *p* = 0.0279, *** *p* ＜ 0.01). Statistical significance was assessed with one-way ANOVA. (C) Relative body weight of GKO mice infected with the two transgenic strains, with mice infected with the *Δcdpk2a* strain showing significantly less weight loss (*p* ＜ 0.05 from 8 DPI to 22 DPI). (D) Survival rates of GKO mice infected with the two transgenic strains (*p* = 0.1492). Statistical significance was assessed with one-way ANOVA.

**Fig 6:**
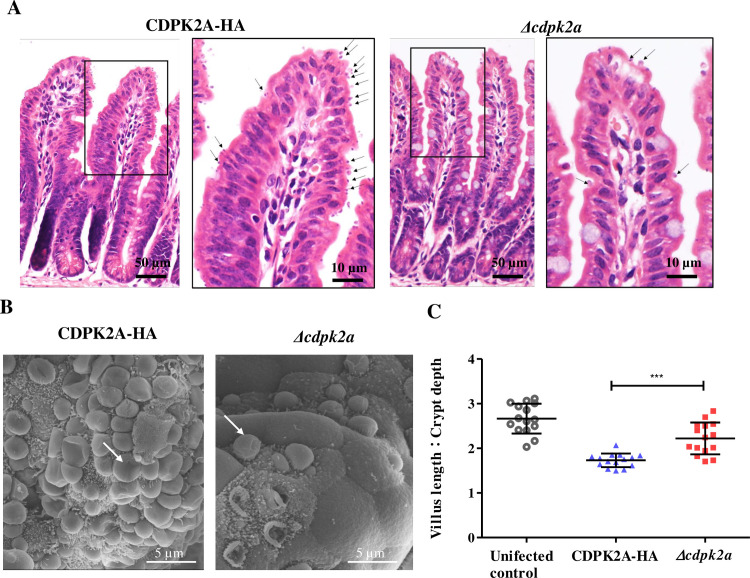
Differences in parasite burden and pathological changes in the ileum of uninfected control GKO mice and GKO mice infected with the CDPK2A-HA and ***Δcdpk2* strains of *Cryptosporidium parvum*.** (A) Histology of the ileum sections at DPI 12 as indicated by hematoxylin and eosin microscopy (original magnification = 400×). (B) Parasite load in the ileum of mice under scanning electron microscopy. (C) Villus length and crypt height ratios from 15 villi in the ileum of uninfected control mice and mice infected with the CDPK2A-HA and *Δcdpk2* strains. *** *p* ＜ 0.01. Statistical significance was assessed with one-way ANOVA.

To assess the importance of CpCDPK2A in the growth and pathogenicity of *C. parvum*, GKO mice were infected with *Δcdpk2a* and CDPK2A-HA strains and compared for oocyst shedding intensity, body weight gain, occurrence of clinical signs, and survival rates. Oocyst shedding in *Δcdpk2a*-infected mice increased much later than in CDPK2A-HA-infected mice, starting at 8 and 4 DPI, respectively (Fig 5A). As a result, the mean OPG at 12 DPI was 3.6 × 10^5^ for Δ*cdpk2a*-infected mice and 9.5 × 10^6^ for CDPK2A-HA infected mice, representing an approximately 25-fold reduction of oocyst shedding intensity in *Δcdpk2a*-infected mice (*p* ＜ 0.01) (Fig 5A). For fecal luminescence activities, the greatest difference between two groups was at 12 DPI, which was 1.9 × 10^5^ for the *Δcdpk2a* group and 4.5 × 10^6^ for the CDPK2A-HA group (*p* ＜ 0.01), representing an approximately 24-fold reduction of infection intensity in *Δcdpk2a*-infected mice (Fig 5B). In addition, mice infected with the *Δcdpk2a* parasites had greater body weight gain and longer survival than those infected with the CDPK2A-HA strain (Fig 5C and 5D). From 8 DPI to 22 DPI, mice infected with the *Δcdpk2a* strain showed significantly less weight loss than those infected with the CDPK2A-HA strain (*p* ＜ 0.05). Between the two infection groups, 50.0% mice infected with the CDPK2A-HA strain died of the infection during 12–24 DPI compared to 16.6% mice infected with the *Δcdpk2a* strain at 30 DPI (*p* = 0.1492). Although the difference in survival rate was not significant, mice infected with the *Δcdpk2a* strain had a longer survival time than those infected with the CDPK2A-HA strain. Two mice in each group were euthanized and parasite load and pathological changes in the ileum tissue were compared among the three groups. HE-stained tissue sections from CDPK2A-HA-infected mice had higher parasite load than those from *Δcdpk2a*-infected mice when examined by light microscopy (Fig 6A). This was confirmed by SEM examination of the ileum tissue (Fig 6B). In addition, compared with *Δcdpk2a* infected mice, CDPK2A-HA-infected mice had more villus blunting and crypt hyperplasia (Fig 6A). This was also confirmed by the measurement of villus height and crypt depth ratio in each mouse, which was significantly lower in CDPK2A-HA-infected animals than in Δ*cdpk2a*-infected animals and uninfected controls (*p* ＜ 0.001; Fig 6C).

## Discussion

Results of the study suggest that CpCDPK2A is expressed across all developmental stages of *C. parvum*, especially in the surface of the motile sporozoites and merozoites We have successfully deleted the *CpCDPK2A* gene using CRISPR-Cas9 and generated viable parasites for *in vitro* and *in vivo* studies, indicating the dispensable nature of CpCDPK2A. Deletion of *CpCDPK2A* did not affect parasite invasion of host cells, but significantly reduced parasite growth *in vitro* and parasite load *in vivo*. These results suggest that CpCDPK2A is involved in both asexual and sexual growth of *C. parvum* and therefore may be a potential target for the development of therapeutics against cryptosporidiosis.

Unlike the limited expression of other CDPKs in particular developmental stages (S2 Table), CpCDPK2A is highly expressed in divergent stages of *C. parvum* (Fig 2). Previously, based on CRISPR/Cas9 methods, CpCDPK1 was expressed only in mature *C. parvum* meronts [5], and CpCDPK5 was expressed during male gamete development of *C. parvum* [12]. Data from the present study suggest that, unlike CpCDPK1 and CpCDPK5, CpCDPK2A is endogenously expressed in all life cycle stages of *C. parvum*. Interestingly, indirect immunofluorescence showed that CpCDPK2A has uneven accumulation on the surface of motile stages of sporozoites and merozoites in *C. parvum* (Fig 2). Similarly, PfCDPK1 was found to be present in the membrane and organelle compartments of blood-stage *Plasmodium parasites* [29]. Despite lacking transmembrane regions, both CpCDPK2A and PfCDPK1 possess several N-myristoylation sites. Myristoylation is known to mediate membrane association and to be involved in stress responsive signaling pathways in plants [30]. Thus, these modification sites are likely to be functionally important in the CpCDPK2A of *C. parvum*.

Similar to CpCDPK5, CpCDPK2A appears to be one of CpCDPKs expressed in the sexual stages of *C. parvum*. Previously, members of the CpCDPKs family were suggested to have different biological functions in *C. parvum* (S2 Table). CpCDPK1, CpCDPK4, CpCDPK6, and CpCDPK9 may be involved in host cell invasion by *C. parvum* [14,16]. CpCDPK3is likely involved in parasite growth. CpCDPK5 is only expressed in the sexual stages of *C. parvum* and is important for the egress of male gametes [12,15,16]. Data from the present study are consistent with previous observations using rCpCDPK2A, which indicated that this protein is expressed in both asexual and sexual stages of *C. parvum* [13]. Using a luciferase assay, we found that deletion of *CpCDPK2A* had no impact on the early asexual development (0–24 HPI) of the parasites, but reduced the growth of the late asexual stage (36 HPI) and sexual stages (36–72 HPI) (Fig 4B). Therefore, CpCDPK2A might contribute to the late phase of asexual replication and growth of sexual stages of *C. parvum*.

Despite the importance of the protein across multiple developmental stages, CpCDPK2A is dispensable in *C. parvum* (Fig 3). Previously, deletion of *CpCDPK1* was shown to be lethal to *C. parvum*, and a conditional knockdown system was necessary to study the function of *CpCDPK1* [10]. Unlike the CpCDPK1 knockout, the *Δcdpk2a* strain in this study remains viable but exhibits delayed development in late asexual and sexual stages and attenuated pathogenicity in mouse models. This phenotype defined CpCDPK2A as a non-essential kinase, but critical for growth and transmission of *C. parvum*. Similarly, recent findings demonstrate that *CpCDPK5* is also dispensable for the survival of *C. parvum*, but leads to less male gametes being released from the microgamont [12]. In *Toxoplasma gondii*, genetic studies have shown that while *TgCDPK1* cannot be disrupted, most other *TgCDPKs* are dispensable [31,32]. This is likely the case for some CpCDPKs in *C. parvum*, as indicated by our gene deletion study of *CpCDPK2A*.

*CpCDPK2A* appears to be a good target to block disease transmission. In previous studies, deletion of noncanonical *TgCDPKs* mostly did not cause phenotypic changes in *T. gondii in vitro* and *in vivo* [31]. In the present study, however, deletion of the *CpCDPK2A* gene has reduced the intensity of oocyst shedding in GKO mice by nearly three logs (Fig 5A). In addition, low parasite loads on the villus surface and nearly normal villus length and crypt depth ratios were observed in mice infected with the *Δcdpk2a* strain (Fig 6). These observations support an important role of this kinase in *C. parvum* development. In recent years, using a conditional protein degradation system, CpCDPK1 has been shown to play a critical role in *C. parvum* proliferation, making it a therapeutic target in *Cryptosporidium* spp. [10]. Several bumped kinase inhibitors have been developed by targeting *CpCDPK1* and have shown good activities against *C. parvum in vitro* and *in vivo* [33]. In addition, CpCDPK5 has been shown to control the production of male gametes and has been also identified as a potential target for blocking disease transmission [12]. Similar to CpCDPK5, CpCDPK2A here may be another potential vaccine target for *C. parvum*. Further studies are required to explore the vaccine potential and action mechanism of CpCDPK2A.

## Methods

### Ethics statement

All experiments with mice were performed in accordance with the guidelines of the Chinese government and the Guide for the Care and Use of Laboratory Animals. The research protocol was reviewed and approved by the Research Ethics Committee of South China Agricultural University (No. 2022C024).

### Maintenance of mice and *C. parvum* isolate

Interferon-γ knockout (GKO) C57BL/6 mice were obtained from the Jackson Laboratory (Bar Harbor, ME, USA) and bred at the Center of Laboratory Animals in South China Agricultural University. They were housed in ventilated isolators under specific pathogen-free (SPF) conditions on a 12:12 light-dark cycle and fed with sterile commercial diet and distilled water.

A *C. parvum* isolate of the IIdA20G1 subtype from a dairy calf during a diarrhea outbreak in Heilongjiang Province, China, was used in the present study [17,18]. The isolate was cloned by a single oocyst infection and propagated in GKO mice every three months. Oocysts were purified from fecal samples of infected mice by sucrose and cesium chloride gradient centrifugation as described [19]. All strains were stored in antibiotics at 4 °C for less than 3 months before use. Free sporozoites were obtained from excysted oocysts by incubation in 0.75% taurodeoxycholic acid (Sigma-Aldrich, Missouri, USA) at 37 °C for 60 min [20]. Intracellular stages of parasites were prepared from HCT-8 (ATCC CCL-244) cultures infected with oocysts for 3–72 hours has described [21]. Free merozoites were collected by centrifugation from the culture medium after HCT-8 cells being infected for 36 hours [22].

### Construction and immunofluorescence detection of the *CpCDPK2A* gene epitope-tagging strain

The *CpCDPK2A* gene of the *C. parvum* isolate was endogenously tagged with 3 × hemagglutinin (3HA) epitope using CRISPR-Cas9 technology as previously described [23]. Briefly, to construct single guide RNA (sgRNA) CRISPR plasmids, sgRNA was selected from a region 203 bp upstream the stop codon in the *CpCDPK2A* (cgd2_1060) gene using the Eukaryotic Pathogen CRISPR Guide RNA/DNA Design Tool (http://grna.ctegd.uga.edu) and inserted into the pACT1:Cas9-GFP and U6:sgTK plasmids to replace the sgTK gene using the CloneExpress II One-Step Cloning Kit (Vazyme, Nanjing, China). To construct the tagging plasmids, the 5′ (204 bp upstream the stop codon) and 3′ (358 bp downstream the stop codon) homologous arms of the *CpCDPK2A* gene were amplified from genomic DNA of *C. parvum*, and the 3HA-Nluc-P2A-neo fragment was amplified from the pINS1–3HA-Nluc-P2A-neo plasmid [24]. The three fragments generated were ligated into plasmid pACT1 using the same cloning kit (Vazyme). After verification by DNA sequence analysis, positive plasmids were extracted using NucleoBond Xtra Midi EF (Macherey-Nagel, Düren, Germany). The *CpCDPK2A*-specific CRISPR plasmid (~50 μg) and tagging plasmid (~50 μg) were co-transfected into 2 × 10^7^ fresh sporozoites using program EH100 in an Amaxa 4D-Nucleofector (Lonza, Basel, Switzerland). The transfectants were selected in three GKO mice with 16 g/L paromomycin in drinking water starting 1 day post infection (DPI) as described [25]. All primers used in the study are listed in S1 Table.

The correct integration of the cassette was confirmed by PCR analysis of fecal samples collected at 12 DPI. The load of transgenic parasites in mice was monitored using the Nano-Glo luciferase assay (Promega, Madison, USA) every 3 days. The expression of CpCDPK2A in developmental stages of *C. parvum* was examined using an immunofluorescence assay (IFA). Briefly, epitope-tagged parasites or cells infected with them on SuperStick coverslips (Waterborne, New Orleans, USA) were fixed in 4% paraformaldehyde in phosphate-buffered saline (PBS), permeabilized with 0.1% Triton-100, and blocked with 3% BSA in PBS. They were labeled with rabbit anti-HA polyclonal antibodies (Cell Signaling Technology, Danvers, USA) as primary antibodies and Alexa Fluor 488-conjugated anti-rabbit IgG (Cell Signaling Technology) as secondary antibodies. The Cy3-labeled polyclonal anti-*C. parvum* antibody Sporo-Glo (Waterborne) and 4′, 6-diamidino-2-phenylindole (DAPI; Beyotime, Shanghai, China) were used to stain *C. parvum* and nuclei, respectively. Images were captured on a BX53 fluorescence microscope (Olympus, Tokyo, Japan) and manipulated using Zen Microscopy software (Carl Zeiss, Oberkochen, Germany). All images were taken with consistent exposure settings, and two independent replicate experiments were conducted to ensure the reliability of the experimental results.

### Construction and assessment of the *CpCDPK2A* gene deletion strain

The *CpCDPK2A* gene deletion strain (*Δcdpk2a*) was constructed using CRISPR-Cas9 technology as previously described [26]. Briefly, to construct double gRNA-CRISPR plasmids, the second guide RNA was selected from a region 150 bp downstream the promoter in the *CpCDPK2A* gene and inserted into the sgRNA-CRISPR plasmid. To construct the deletion plasmid, the plasmid pINS1–3HA-Nluc-P2A-neo was used as a template to amplify the Nluc-P2A-neo fragment, while the 5′ (900 bp before the promoter) and 3′ (900 bp after the stop codon) homologous arms of the *CpCDPK2A* gene were amplified from *C. parvum* genomic DNA as described above. Subsequently, the three fragments were engineered into the plasmid pACT1 using the CloneExpress II One-Step Cloning Kit (Vazyme). The gRNA-CRISPR plasmids (~50 μg) and the deletion plasmid (~50 μg) were mixed and co-transfected into fresh sporozoites of *C. parvum* as described above. The selection and PCR analysis of transfectants and determination of the parasite load in infected mice were performed as described above.

The lack of CpCDPK2A expression in *Δcdpk2a* parasites was confirmed by IFA using polyclonal and monoclonal antibodies against recombinant CpCDPK2A (rCpCDPK2A) prepared in a previous study [13]. The growth rate of the CDPK2A-HA strain and *Δcdpk2a* strain in HCT-8 cells at 3, 12, 24, 36, 48, 60, and 72 hour post infection (HPI) was compared using the luciferase assay described above. Each measurement was performed twice in four replicates and data from the two groups were compared using an unpaired *t* test (GraphPad software 5.0,http://www.graphpad.com). All strains were stored in antibiotics at 4 °C for less than 3 months before use.

### Evaluation of pathogenicity of *Δcdpk2a* strain in vivo

To evaluate the effect of the *CpCDPK2A* gene deletion on *C. parvum* pathogenicity, 6-week-old GKO mice (6 mice per group) were orally infected with the *Δcdpk2a* and CDPK2A-HA strains (1,000 oocysts/mouse), with six mice being used as uninfected controls. All strains stored in antibiotics at 4 °C for less than 3 months before use. Oocyst shedding intensity, body weight, clinical signs and mortality were recorded to evaluate the pathogenicity of parasites. Both the luciferase assay and *SSU* rRNA gene-based qPCR were used to assess the parasite load. The latter was performed in a LightCycler 480 (Roche, Basel, Switzerland), with the number of oocysts per gram of feces (OPG) being calculated based on threshold cycle (C_T_) values using a standard curve [27].

Histological examinations and scanning electron microscopy (SEM) were used to further compare the parasite load and virulence of the CDPK2A-HA and *Δcdpk2a* strains. For this, the ileum was collected at 12 DPI from mice infected with CDPK2A-HA and *Δcdpk2a* strains and control mice, fixed in 4% paraformaldehyde for 24 h, and processed for sectioning and hematoxylin-eosin (HE) staining using routine procedures. The stained tissue sections were examined under an BX53 light microscope (Olympus). Random images were captured at 400 × magnification to measure villus length and crypt depth (*n* = 15 villi per mouse) using Image J (http://imagej.nih.gov/ij/) as described previously [28]. Some tissue samples were fixed in 2.5% glutaraldehyde and 1% osmium tetroxide and processed for SEM under an EVO MA 15/LS 15 (Carl Zeiss Microscopy GmbH, Jena, Germany) [17].

## Supporting information

S1 Fig Verification of the *CpCDPK2A* gene deletion using immunofluorescence microscopy with polyclonal antibodies against recombinant CpCDPK2A (rCpCDPK2A). Wild type (WT) and *Δcdpk2a* parasites were stained with anti-rCpCDPK2A polyclonal antibodies (in green) and anti-*Cryptosporidium* antibodies (in red), with the nuclei being counter-stained with DAPI (in blue). Scale bars = 2 μM.
(TIF)

S1 Table All primers used in the study are listed in S1 Table. (PDF)

S2 Table Comparative overview of calcium-dependent protein kinases (CDPKs) in *Cryptosporidium parvum.* (XLSX)

S1 Raw Data Raw data for all figures in this study. (XLSX)
