# Supplementary figures and images for: Calcium-dependent protein kinases 2A involved in the growth of both asexual and sexual stages of Cryptosporidium parvum
Source: PLoS Negl Trop Dis. 2025 May 28;19(5):e0013107. doi: 10.1371/journal.pntd.0013107 (PMC12119106; doi:10.1371/journal.pntd.0013107)

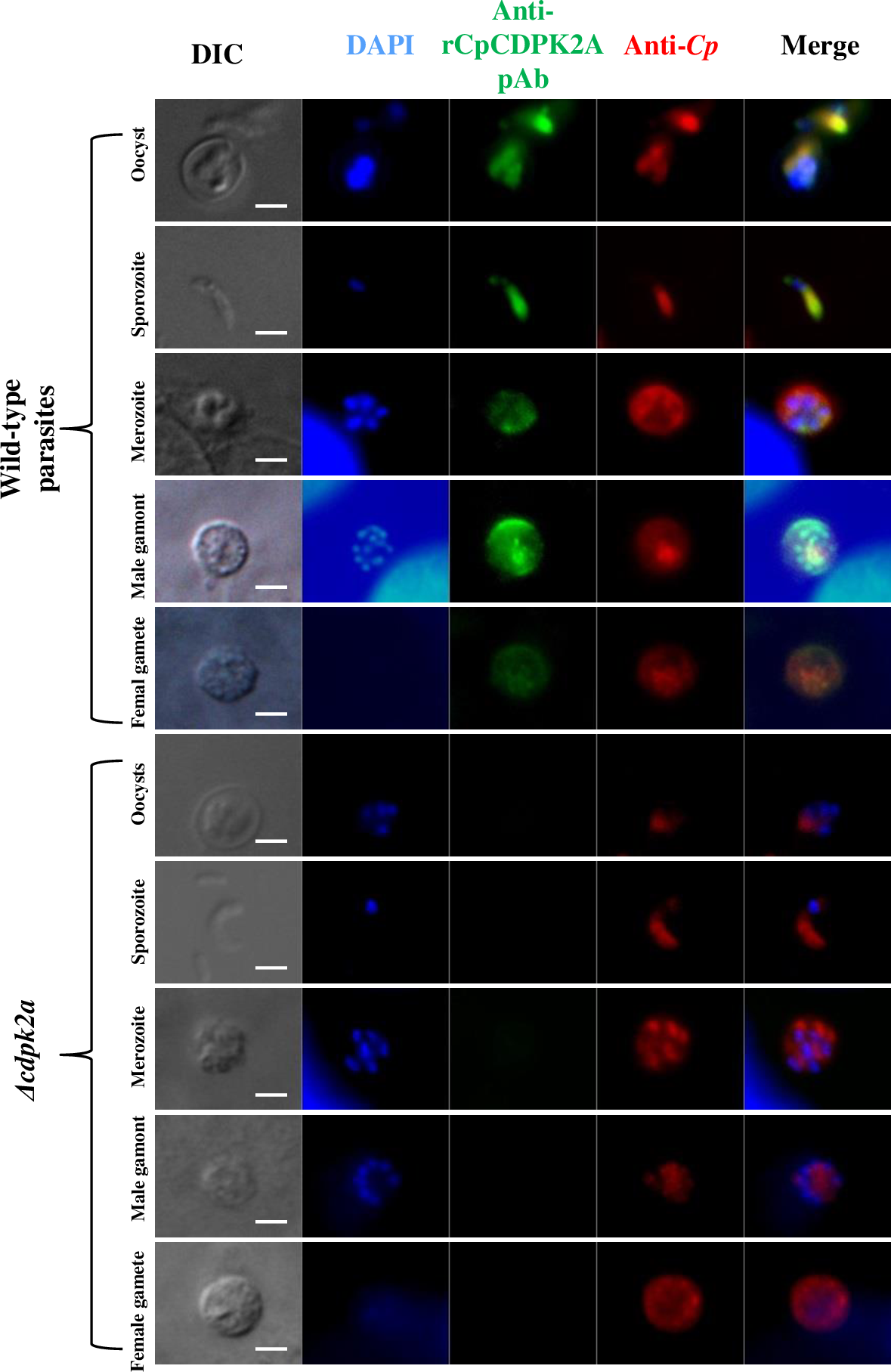

Supplement: S1 Fig — Wild type (WT) and Δcdpk2a parasites were stained with anti-rCpCDPK2A polyclonal antibodies (in green) and anti-Cryptosporidium antibodies (in red), with the nuclei being counter-stained with DAPI (in blue). Scale bars = 2 μM. (TIF) [file pntd.0013107.s001.tif]
