# Supplementary material for: Calcium-dependent protein kinases 2A involved in the growth of both asexual and sexual stages of Cryptosporidium parvum
Source: PLoS Negl Trop Dis. 2025 May 28;19(5):e0013107. doi: 10.1371/journal.pntd.0013107 (PMC12119106; doi:10.1371/journal.pntd.0013107)
Supplement: S1 Table — (PDF) [file pntd.0013107.s002.pdf]

Table S1 – Oligonucleotides used in this study.

Usage	Oligo name	Sequence (5' – 3')	Specific purpose	Source
Tagging	CDPK2A-C ter-F	AGTGAATTCGAGCTGAGCTCGAATAGCGGGATTACTGAG	Amplify *cdpk2a C-terminus* homology arm (395 bp) for building pCDPK2A-3HA-Nluc-P2A-neo by Gibson assembly	This study; IDT
	CDPK2A-C ter-R	GCCCGAGCCCTTGCTAGCAGAATTAGATCTTCTGAACATTTCCA		This study; IDT
	CDPK2A-3UTR-F	TTGACGAATTCTTCTGATTAATAGCCTTTTATTATCACTTAATATTCC	Amplify *cdpk2a 3'UTR* homology arm (236 bp) for building pCDPK2A-3HA-Nluc-P2A-neo by Gibson assembly	This study; IDT
	CDPK2A-3UTR-R	GTCGACTCTAGAGGATCTCCGGAACATCAACTAATATTAGAAACAT		This study; IDT
	Nluc-F1	GGAGATCCTCTAGAGGTCGAC	Amplify *plasmid backbone* (2649 bp) for building pCDPK2A-3HA-Nluc-P2A-neo by Gibson assembly	This study; IDT
	Nluc-R1	GAGCTCAGCTCGAATTCAC		This study; IDT
	pUC19-F1	GCTAGCAAGGGCTCGGGC	Amplify 3HA-Nluc-P2A-neo casstte (2048 bp) for building pCDPK2A-3HA-Nluc-P2A-neo by Gibson assembly	This study; IDT
	PUC19-R1	CTTCTTGACGAATTCTTCTGATTA		This study; IDT
	tracr RNA-R1	GTTTTAGAGCTAGAAATAGCAAG	Amplify Cas9 *plasmid backbone* (9963 bp) for building pACT1:Cas9-GFP, U6:sgCDPK2A	This study; IDT
	U6 F	CCCAACACTTAACCTTTCAGT		This study; IDT
	CDPK2A-gRNA1-linker	CTGAAAGGTTAAGTGTTGGGGCAAAGGAGAAGTCGGATCTGGAGTTTTAGAGCTAGAATAGC	Building pACT1:Cas9-GFP, U6:sgCDPK2A by Gibson assembly	This study; IDT
Deletion	CDPK2A-5'UTR-F1	AGTGAATTCGAGCTGAGCTCTAAAGGGGTAAACAAGCAAAG	Amplify *cdpk2a 5'UTR* homology arm (900 bp) for building pCDPK2A-Nluc-P2A-neo-CDPK2A by Gibson assembly	This study; IDT
	CDPK2A-5'UTR-R1	GTATATTTAGTTTCCCCACCTCTCCTTTTACGAAAAAGCAT		This study; IDT
	INS1-3'UTR-F2	TTCTTGACGAATTCTTCTGAATAGCCTTTTATTATCACTTAATATTCC	Amplify *cdpk2a 3'UTR* homology arm (900 bp) for building pCDPK2A-Nluc-P2A-neo-CDPK2A by Gibson assembly	This study; IDT
	INS1-3'UTR-R2	GTCGACTCTAGAGGATCTCCTTTAGTATTCAGCAATACTGAGAC		This study; IDT
	Nluc-F2	GGAGATCCTCTAGAGTCGAC	Amplify mCh-*Nluc-P2A-neo casstte* (1700bp) for building pCDPK2A-Nluc-P2A-neo-CDPK2A by Gibson assembly	This study; IDT
	Nluc-R2	GAGCTCAGCTCGAATTCAC		This study; IDT
	pUC19-F2	AGGTGGGGAAACTAAATATACTGAAATTCG	Amplify plasmid backbone (2649 bp) for building pCDPK2A-Nluc-P2A-neo-CDPK2A by Gibson assembly	This study; IDT
	PUC19-R2	TCAGAAGAATTCGTCAAGAAGACGATAGAAG		This study; IDT
	CDPK2A-gRNA2-linker	CTGAAAGGTTAAGTGTTGGGGAGGTTGATGTAGATGGAGAGTTTTAGAGCTAGAAATAGC	Building pACT1:Cas9-GFP, U6:sgCDPK2A-2 by Gibson assembly	This study; IDT
	gRNA-line-F	GTCATAGCTGTTTCCTGTGTGAAATTG	Primer set used to amplify ACT1:Cas9-GFP, U6:sgCDPK2A casstte (9983 bp) for building pACT1:Cas9-GFP, dual, U6:sgCDPK2A-KO by Gibson assembly	This study; IDT
	gRNA-line-R	GCTTGCATGCCTGCATAGTATATCC		This study; IDT
	Cassettet-gRNA2-F	CTATGCAGGCATGCAAGCTTCCAGTGAATTCGAGCGCC	Amplify U6:sgCDPK2A-2 casstte (878 bp) for building pCRISPR-dual-CDPK2A by Gibson assembly	This study; IDT
	Cassettet-gRNA2-R	ACAGGAAACAGCTATGACCCAAATTTCTCCAACCAGT		This study; IDT
PCR genotyping	CDPK2A-C ter-F1 (P1)	GGCTTTATGAAAACAGGCATAG	Primer set "5' Ins" used to detect CDPK2A-3HA insection site<br>WT = 460 bp; tagging C-ter= 882 bp; tagging N-ter= 680 bp;	This study; IDT
	CDPK2A-C ter-R1 (P2)	TCGGCAGACTTAACCAGCTA		This study; IDT
	CDPK2A-N ter-F1 (P3)	GCTGAAGAACTTGGTGGTGA	Primer set "3' Ins" used to detect CDPK2A-3HA insection site<br>WT = 460 bp; tagging C-ter= 882 bp; tagging N-ter= 680 bp;	This study; IDT
	CDPK2A-N ter-R1 (P4)	GCTGCAATTATCAAGCTCTCAC		This study; IDT
	WT-F1	GGGAATACAGGAACAAGGCT	Primer set "WT"<br>WT = 460 bp; tagging C-ter= 882 bp; tagging N-ter= 680 bp;	This study; IDT
	WT-R2	GTTCTCCTCCTGTGTAAAGC		This study; IDT
	CDPK2A-C ter-F2 (P5)	TATCGAACGTGCTCGTACTCGTATGGAA	Primer set "5' Ins" used to detect Δ*ins1* spanning the 5' CRISPR targeting site<br>WT = 2100 bp; tagging C-ter= 1241 bp; tagging N-ter= 1739 bp;	This study; IDT
	CDPK2A-C ter-R2 (P6)	TCTCTTGCTCAATTCTTATTCACTTTAG		This study; IDT
	CDPK2A-N ter-F2 (P7)	ATTCTTCCCAAAATGACTTGCTCTGACC	Primer set "3' Ins" used to detect Δ*ins1* spanning the 3' CRISPR targeting site<br>WT = 2100 bp; tagging C-ter= 1241 bp; tagging N-ter= 1739 bp;	This study; IDT
	CDPK2A-N ter-R2 (P8)	CCACTAATTCATATTCCCGCCCTTAGAG		This study; IDT
	WT-F2	ATGGGAAATCTGCAGTAGGGAATAC	Primer set "WT"<br>WT = 2100 bp; tagging C-ter= 1241 bp; tagging N-ter= 1739 bp;	This study; IDT
	WT-R2	GCAGAATTAGATCTTCTGAACATTTCC		This study; IDT
